# Solid-Phase Synthesis of the Lipopeptide Myr-HBVpreS/2-78, a Hepatitis B Virus Entry Inhibitor

**DOI:** 10.3390/molecules15074773

**Published:** 2010-07-07

**Authors:** Alexa Schieck, Thomas Müller, Andreas Schulze, Uwe Haberkorn, Stephan Urban, Walter Mier

**Affiliations:** 1 Department of Nuclear Medicine, University Hospital Heidelberg, Im Neuenheimer Feld 400, 69120 Heidelberg, Germany; 2 Department of Infectious Diseases, University Hospital Heidelberg, Im Neuenheimer Feld 350, 69120 Heidelberg, Germany

**Keywords:** solid phase synthesis, difficult sequences, hepatits B virus entry inhibitor

## Abstract

Chronic HBV infection is the leading cause of liver cirrhosis and hepatocellular carcinoma (HCC). Synthetic peptides derived from the *N*-terminus of the large HBV envelope protein (L-protein) have been shown to efficiently block HBV entry. Myr-HBVpreS/2-78, the parent compound of these drugs, inhibits human HBV infection *in vitro* and *in vivo*. An efficient synthesis is required, as these peptides constitute a novel class of anti HBV drugs. Consequently, the solid phase synthesis of the *N*-terminal 77 amino acids of the viral L-protein was studied in detail. The peptide was *N*-terminally myristoylated to resemble the natural, postranslationally modified protein. The synthesis was monitored using the Fmoc cleavage pattern of the solid phase synthesis on a standard peptide synthesizer and by LC-MS analyses of the arising side products. “Difficult sequences” in the positions 42-47 of the peptide sequence complicate the efficient synthesis of the 77-mer peptide HBVpreS/2-78. Attempts were undertaken to optimize the synthesis by heating, double coupling or the use of pseudoproline dipeptides. HPLC-MS analyses showed that the efficiency of the synthesis could be increased best by temperature elevation. This resulted in a higher purity of the crude product after solid phase synthesis. It was possible to minimize the occurrence of side products due to the positive effects related to higher reaction temperature. In conclusion, the peptide is accessible by stepwise SPPS without the necessity of segment coupling.

## 1. Introduction

The human hepatitis B virus (HBV) is a small, enveloped DNA virus that causes acute and chronic liver infection. It is assumed that 2 billion people worldwide have had contact with that virus [1]. According to recent data, about 360 million people are living with a chronic HBV infection. Due to this chronic infection the probability to develop liver cirrhosis or hepatocellular carcinoma is increased [2]. After smoking, the chronic hepatitis B infection is the second main exogen risk factor to develop liver cancer or a hepatocellular carcinoma (HCC). As a consequence each year about one million people die because of the long-lasting liver damage.

**Figure 1:**
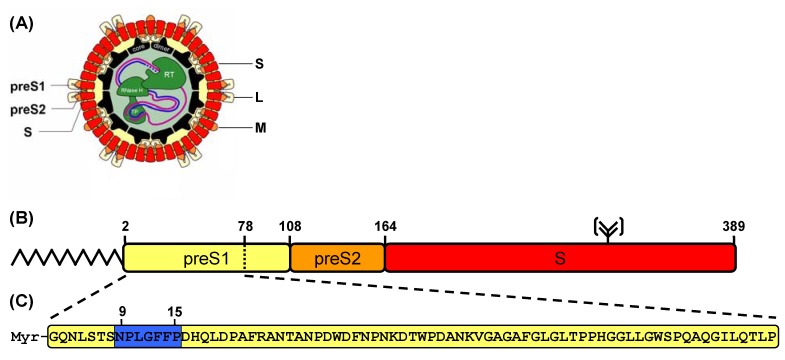
(a) The human hepatitis B virion. The partially double stranded DNA genome is encapsulated by an icosahedral shell and surrounded by three different surface proteins (L, M and S). (b) Domain structure of the large (L) HBV envelope protein. The L-protein consists of three subdomains, the preS1-domain (108 aa, yellow), the preS2-domain (55 aa, orange) and the S-domain (226 aa, red). The L-protein is *N*-terminally myristoylated at position 2 of the peptide sequence. Within the S-domain the protein bears a potential *N*-glycosylation site at Asn-309. (c) Acylated peptide sequence derived from the HBV L-protein that specifically inhibits a HBV infection *in vitro* and *in vivo* by blocking the virus entry. The myristoylated peptide corresponding to the *N*-terminal 77 aa of the preS1-part of the HBV L-protein is shown. The amino acids underlined in blue define the pharmacophore of the peptide sequence [3,9].

Present therapeutic regimens for HBV suppress either the host immune system by interferon-α or inhibit the reverse transcription of the viral pregenomic RNA by nucleoside inhibitors (lamivudine, adefovir, entecavir). Both strategies show limitations as they are non-curative and in the case of nucleoside analoga the application provoke the development of drug-resistant virus strains. Looking for new antiviral strategies, we have recently demonstrated that acylated peptides derived from the HBV envelope block the hepatitis B virus entry *in vitro* [3,4,5] and *in vivo* [6]. Myr-HBVpreS/2-78, the parent compound of these peptides, is a 77 amino acid peptide representing the *N*-terminal part of the viral L-protein. The peptide is myristoylated on the *N*-terminus and amidated at the *C*-terminus (Figure 1). As the region 3-77 of the viral L-protein is required for HBV infectivity in primary human hepatocytes [7,8], the corresponding peptide is expected to address a cellular receptor. Further structure–activity relationship (SAR) studies revealed that the amino acids 9 to 15 define the pharmacophore of the peptide sequence. In addition to the lipidic moiety at the *N*-terminus this sequence motif is mandatory for the inhibitory effect [3,9]. The inhibition of the viral HBV infection by preventing the virus entry on hepatocytes shows a new therapeutic strategy. Consequently, these acylated peptides belong to a class of entry inhibitors and provide a new option to treat chronic HBV infections.

The first representative of antiviral entry inhibitors was T-20 (Fuzeon), a peptide consisting of 36 amino acids that is able to block the entry of HIV into human cells. In general, peptides have a huge potential as drugs as they represent bioactive structures inside biological systems (“Natural Pharmaceuticals”). They are smaller than proteins and therefore in general less immunogenic. Today, more than 60 approved peptide pharmaceuticals are on the market, another 200 peptides are in clinical development [10,11].

For the commercial production of peptides, chemical synthesis is still the most universal approach, since it permits a straight forward access to all possible sequences. With the use of unnatural amino acids that are completely synthetic in origin or pseudo-peptide bonds, a much wider chemical diversity is possible than peptide derivatives produced by recombinant technologies. Most short peptides can be obtained by standard stepwise solid-phase synthesis (SPPS) [12,13]. Carefully optimized SPPS protocols generate a wide range of peptides, efficiently synthesized in a fully automated process using commercially available peptide synthesizers and the mild Fmoc/*t*Bu-strategy [14,15]. Nevertheless there are peptides difficult or impossible to obtain because of the existence of “difficult sequences” within the peptide chain. The difficulties associated with these sequences are mainly related to intra- and/or intermolecular aggregation, secondary structure formation, and steric hindrance of protecting groups. Consequently, the deprotected α-amino group of the growing peptide chain is only partly accessible for amide bond formation and the possibility to generate premature termination of the sequence is increased [16]. Many attempts have been undertaken for the prediction and prevention of such difficult sequences.

The present article describes our attempts to synthesize the lipopeptide Myr-HBVpreS2-78 which consists of 77 amino acids. The comparison of the stepwise SPPS synthesis and a prediction of the difficulties within the peptide sequence, are shown. Difficult sequences were identified by using a software [17] based on the Chou and Fasman secondary structure prediction algorithm [18]. Based on the identification of difficult synthetic sections in the target sequence it was possible to optimize the synthesis by identifying the side products by LC-MS analyses. Though the use of pseudoproline dipeptides and additional coupling steps (double couple) could not minimize the occurrence of side products, it was possible to optimize the synthesis applying an elevated temperature of 50 °C during the solid phase synthesis.

## 2. Results and Discussion

For subsequent modifications tyrosine and lysine were added to the *C*-terminal end of the peptide chain. The lysine derivative was orthogonally protected by ivDde to enable a specific labelling of the peptide at the ε-amino group. 

**Figure 2:**
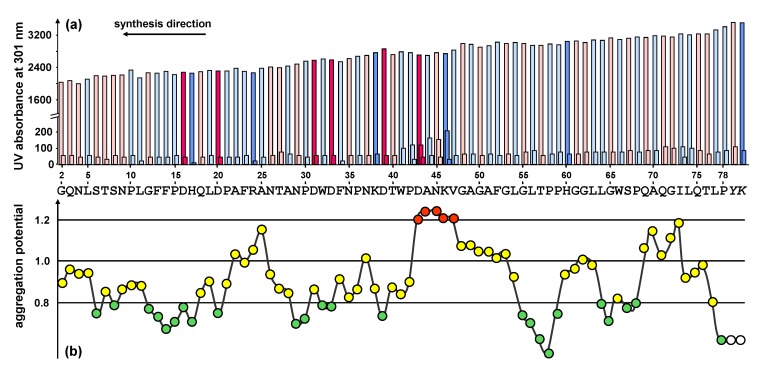
(a) UV measurement (Fmoc cleavage pattern) of the peptide synthesizer during the standard stepwise synthesis of HBVpreS/2-78. The value for the second deprotection step is shown in a fourfold enhancement. Colours used for the different amino acids: rose (polar), light blue (unpolar), pink (acidic) and blue (basic). A slight gradual decrease of the Fmoc cleavage value was observed. In the region from aa 42 to aa 46 an additional deprotection step was necessary. (b) Prediction of synthetically difficult sequences according to the aggregation potential of the growing peptide chain [17]. A graphical representation of the aggregation potential for amino acids starting from the third position of the peptide chain is presented. The sequence shows potential difficult couplings after two or three consecutive potentials have exceeded the value 1.1: the peptide chain tends to aggregate resulting in a slower aminoacylation. For the sequence motif presented, the program shows difficulties within the positions 43 to 47 of the peptide sequence (shown in red).

To predict potentially difficult regions of the peptide, the sequence was analyzed using the Peptide Companion software package [17]. The analysis of the aggregation potential revealed that the sequence is prone to be difficult from position 43 to 47 (DANKV) of the peptide sequence. Minor difficulties were predicted at position 25 and in the very beginning of the synthesis at the positions 70 to 73 (Figure 2b).

The first exploratory attempt to synthesize the peptide HBVpreS/2-78 was made by standard stepwise solid-phase synthesis using the Fmoc/tBu strategy. The Fmoc cleavage pattern of the Applied Biosystems 433A peptide synthesizer revealed the necessity of additional deprotection steps in the region from 42 to 46 of the peptide sequence (Figure 2a). The changes of the Fmoc cleavage signal are in perfect agreement with the predicted structured properties and provide an indicator for significant aggregations of the growing peptide.

**Figure 3:**
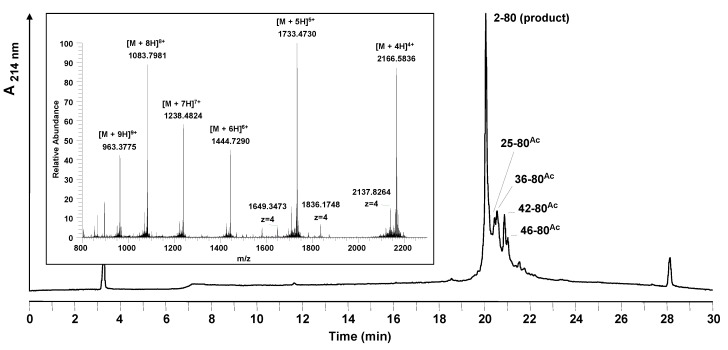
Analytical HPLC and mass spectrometric analysis of the crude peptide after standard stepwise solid phase synthesis at room temperature. The main peak of the HPLC chromatogram corresponds to the desired product. Besides the product, side products were identified by LC-MS. All of the side products are truncated acetylated sequences; their sequences are marked in the chromatogram. Conditions: Hypersil Gold C_18_ column; linear gradient of 0.1% TFA in water to 0.1% TFA in acetonitrile in 30 minutes, flow rate of 0.2 mL/min, 60 °C, wavelength of 214 nm. Insert: mass spectrum obtained for the main peak (19.9–20.4 min).

Mass spectrometry analysis of the crude peptide products showed a number of terminated peptide by-products all blocked with a N^α^-acetyl group (Figure 3). No source of the acetyl group had been used during the synthesis: capping was not performed and neither acetic acid nor acetic anhydride was used in the synthesis reagents. A comparison of the difficult sequence prediction with the results obtained by solid phase synthesis show that the acetylated by-products arise in the regions identified to have a higher aggregation potential.

Truncations and acetylations were detected when Fmoc-Arg(Pbf), Fmoc-Asn(Trt) and Fmoc-Trp(Boc) were coupled. For arginine a correlation of the amino acid used to the appearance of acetylated by-products could be observed. In the case of asparagine and tryptophan only at the positions pointed out acetylated truncation sequences could be detected. At positions 4, 9, 26, 29, 35, 37 (Asn) and 32 and 66 (Trp) of the peptide sequence no acetylation occurred, although the same amino acids were applied.

We hypothesized that free acetic acid was present in the Fmoc-amino acids and induced capping in the difficult regions of the peptide synthesized. ^1^H-NMR of the building block used confirmed this by direct observation of the acetic acid in a sample of Fmoc-Asn(Trt) used in this experiment. The tendency for aggregation/folding depends critically on the nature of the peptide chain with sequences containing a high proportion of Ala, Val, Ile, Asn, or Gln residues showing the highest propensity for aggregation effects [19,20,21]. The findings observed show that due to the higher aggregation potential the coupling reaction to the next amino acid is hindered. Possible side reactions such as the reaction of acetic acid are prefered.

**Figure 4:**
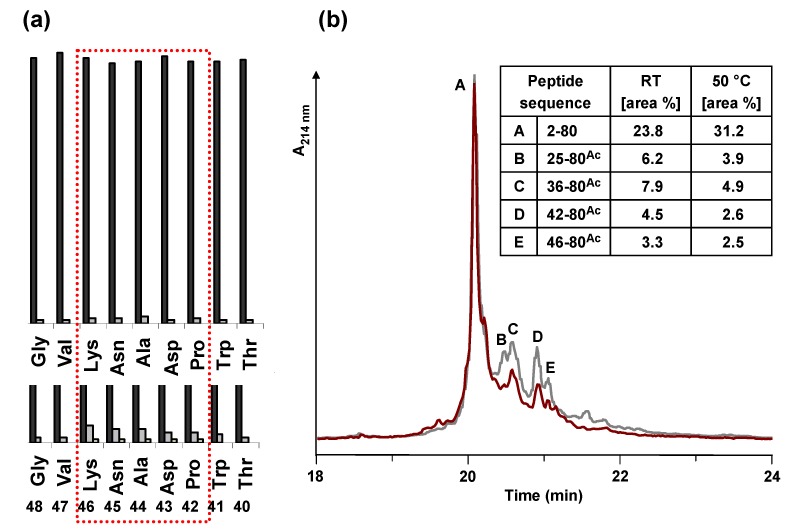
(a) Comparison of the UV measurement (Fmoc cleavage pattern) of the peptide synthesizer during the synthesis of HBVpreS/2-78 at room temperature (RT) and 50 °C. Compared to the synthesis at RT (lower part), during the synthesis at elevated temperature no third deprotection step in the region from aa 42 to aa 46 of the peptide sequence was required. (b) Analytical HPLC of the crude peptide after solid phase synthesis at elevated temperature. HPLC chromatogram of the product and major side product region (red line). The main peak corresponds to the desired product. In comparison to the synthesis at room temperature (grey line), the side products are decreased. Integration results (area %) of the two HPLC chromatograms are presented in the table. Conditions: Hypersil Gold C_18_ column; linear gradient of 0.1% TFA in water to 0.1% TFA in acetonitrile in 30 minutes, flow rate of 0.2 mL/min, 60 °C, wavelength of 214 nm.

Different strategies were pursued to overcome the difficulties within the synthesis. It was tried to increase the coupling yield by double coupling [16] of the amino acid and using HATU instead of HBTU. To avoid a truncation of the peptide at position 35 of the peptide sequence, the pseudoproline dipeptide Fmoc-Asp(OtBu)-Thr(Ψ^Me,Me^pro)-OH was used at position 40 of the peptide sequence. The dipeptide building blocks have a proline-like ring structure and therefore act like proline as a structural disruptor [22]. Analytical HPLC and mass spectrometric analysis of the crude peptide after solid phase synthesis showed that none of these strategies could enhance the efficiency of the peptide synthesis – an almost identical pattern of side products was detected. 

The attempts described so far were carried out at room temperature. However, significant improvements for difficult peptide sequences were obtained in several cases by performing peptide coupling steps at elevated temperatures (30–80 °C) [23,24,25]. Consequently, the solid phase synthesis of HBVpreS/2-78 was performed by heating the reaction vessel during synthesis. A self made heating unit was used and operated at 50 °C. The control of the heating unit was integrated into the software of the Applied Biosystems 433A peptide synthesizer resulting in a variable control of the heating appliance. In the regions from 23–28 and 35–51 of the peptide sequence the reaction vessel was heated, the remaining regions were synthesized at room temperature. By comparing the crude product obtained after heating with the synthesis at room temperature it could be demonstrated that the formation of almost all side products was decreased. Under the improved conditions the peptide could be obtained in higher yields and in a higher purity (Figure 4).

**Figure 5:**
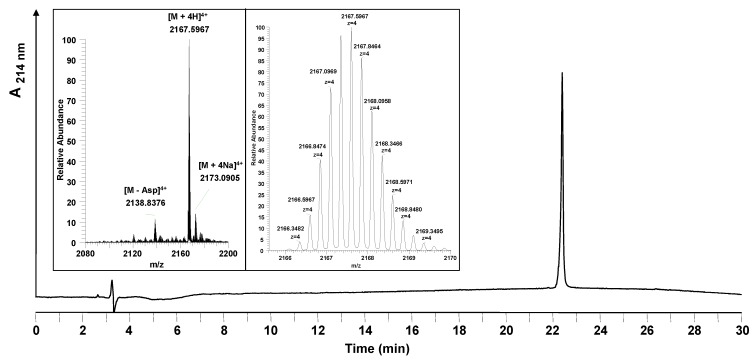
Analytical HPLC and mass spectrometric analysis of the purified entry inhibitor Myr-HBVpreS/2-78. The HPLC chromatogram shows one single peak corresponding to the purified peptide. Insert: mass spectrum obtained (22.37–23.05 min). The signals obtained show the 4-fold charged m/z signals belonging to the desired peptide. The exact monoisotopic mass was calculated to be [M+4H]^4+^ = 2166.3504 (measured: [M+4H]^4+^ = 2166.3487). Besides the product, one side product was identified to be a peptide missing an aspartic acid in the peptide sequence ([M+4H]^4+^ = 2137.5931). Conditions: Hypersil Gold C_18_ column; linear gradient of 0.1% TFA in water to 0.1% TFA in acetonitrile in 30 minutes, flow rate of 0.2 mL/min, 60 °C, wavelength of 214 nm.

The coupling of myristic acid to the *N*-terminus resulted in a shift of the main HPLC-peak showing that the reaction was almost quantitative. The acetylated side products remained at the starting position (data not shown). The myristoylated Myr-HBVpreS/2-78 was purified by preparative HPLC. As Figure 5 shows, the HPLC chromatogram after purification consists of a single product peak. The analysis of the peptide synthesized by LC-MS confirmed formation of the desired product. The resulting purity of Myr-HBVpreS/2-78 defined after HPLC analysis was found to be ≥98%. The overall yield was 7.3%.

## 3. Experimental

### 3.1. Materials

All reagents and solvents were of standard quality and used without further purification unless indicated. 9-Fluorenylmethoxycarbonyl (Fmoc)-protected L-α-amino acids were purchased from Bachem Distribution Services (Weil am Rhein, Germany). 1-[Bis(dimethylamino)methylene]-1*H*-benzo-triazolium hexafluorophosphate 3-oxide (HBTU), piperidine, diisopropylethylamine (DIPEA) and trifluoroacetic acid (TFA) were obtained from Biosolve (Valkenswaard, Netherlands). *N*-methyl-pyrrolidone (NMP) was obtained from Applied Biosystems (Darmstadt, Germany). Triisopropylsilane (TIS) was purchased from Sigma Aldrich (Steinheim, Germany) and myristic acid was obtained from ICN Biomedicals (Ohio, USA). TentaGel R RAM resin with a substitution of 0.19 mmol/g was purchased from Rapp Polymere (Tübingen, Germany).

### 3.2. Methods

#### 3.2.1. Peptide synthesis

Peptide synthesis was performed on an Applied Biosystems 433A peptide synthesizer using Fmoc/*t*Bu chemistry. Peptides were synthesized in a 0.05 mmol scale using a 10-fold molar excess of Fmoc-protected amino acids (0.5 mmol), activated by a 10-fold excess of HBTU in the presence of DIPEA (2 M). N^α^-Fmoc protecting groups were removed by treating the resin-attached peptide with piperidine (20% v/v) in NMP.

#### 3.2.2. Deprotection and Cleavage

The protected peptide was cleaved from the solid support by treatment with TFA/TIS/H_2_O = 95:2.5:2.5 for two hours. The cleaved product was precipitated in diethyl ether and centrifuged at 2500 rpm for 5 minutes. The pellet was washed by resuspension in diethyl ether and centrifugation. The washing step was repeated three times.

#### 3.2.3. HPLC analysis

Analytical reversed-phase HPLC (RP-HPLC) analyses were performed on an Agilent 1090 HPLC system using an Atlantis T3 C_18_ column (0.46 × 150 mm). Retention times are given for gradient elution under the following conditions: 0.1% TFA in water to 0.1% TFA in acetonitrile in 40 minutes; flow rate 0.5 mL/min; 50 °C; absorbance λ = 214 nm.

#### 3.2.4. Mass spectrometry

A mass spectrometer supporting orbitrap technology (Exactive, Thermo Fisher Scientific) was used to analyse the peptides synthesized. As HPLC system served an Agilent 1200 system using a Hypersil Gold C_18_ column (0.21 × 200 mm). A mixture of caffeine, MRFA and Ultramark 1621 was used for mass calibration in the positive-ion mode. Full scan single mass spectra were obtained by scanning from m/z = 200-4,000 for 30 minutes. HPLC conditions were the following: linear gradient of 0.1% TFA in water to 0.1% TFA in acetonitrile in 30 minutes, flow rate 200 µL/min, 60 °C, absorbance λ = 214 nm.

### 3.3. Synthesis of HBVpreS/2-78

The assembly of the sequence was performed as described above. All side chain protecting groups were TFA-labile, except for 1-(4,4-dimethyl-2,6-dioxocyclohexylidene)-3-methylbutyl- (ivDde) on lysine at position 80 of the peptide sequence. Retention time of a test cleavage sample (analytical HPLC): 23.3 min. ESI-MS: m/z = 2165.3296 [M+4H]^4+^, 1732.4706 [M+5H]^5+^, 1443.8946 [M+6H]^6+^, 1237.7680 [M+7H]^7+^; 1083.1746 [M+8H]^8+^, mean value: 8657.3156 g/mol (M_calc_: 8657.3048 g/mol).

### 3.4. Synthesis of Myr-HBVpreS/2-78

Myristic acid was coupled to the *N*-terminal end of HBVpreS/2-78. A solution of myristic acid (4 eq.), HATU (4 eq.) and DIPEA (60 µL) in DMF was added to the resin. The reaction mixture was shaken for 4 h, afterwards the resin was washed with DMF (3 × 1 mL) and DCM (3 × 1 mL). Retention time of a test cleavage sample (analytical HPLC): 26.8 min. ESI-MS: m/z = 2217.8760 [M+4H]^4+^, 1774.5046 [M+5H]^5+^, 1478.9204 [M+6H]^6+^, 1267.7914 [M+7H]^7+^; 1109.4434 [M+8H]^8+^, mean value: 8867.4800 g/mol (M_calc_: 8867.5032 g/mol). For removal of the ivDde group, a solution of 2% hydrazine monohydrate in DMF (v/v) was added to the resin swollen in DMF. The mixture was shaken at room temperature for 5, 7, 10 and 15 minutes. Each time the solution was removed and replaced by a fresh solution of 2% hydrazine monohydrate in DMF. The resin was washed with DMF (3 × 1 mL), neutralized with 10% DIPEA in DMF (3 × 1 mL), washed with DMF (3 × 1 mL), DCM (3 × 1 mL) and dried *in vacuo*. The peptide was cleaved from the dried resin and dissolved in water/acetonitrile for purification. Preparative RP-HPLC separation was performed on a Gilson / 321 pump HPLC system. For purification the following conditions were used: Chromolith^®^ SemiPrep-column (10 × 100 mm); gradient elution from 0.1% TFA in water to 0.1% TFA in acetonitrile/water = 70/30 over 10 minutes; flow rate 10 mL/min; absorbance λ = 214 nm. The retention time of Myr-HBVpreS/2-78 was 7.8 min. Retention time of the purified peptide (analytical HPLC): 25.2 min. ESI-MS: m/z = 2166.3487 [M+4H]^4+^, 1733.2818 [M+5H]^5+^, 1444.5702 [M+6H]^6+^, 1238.3465 [M+7H]^7+^; 1083.6782 [M+8H]^8+^, mean value: 8661.3684 g/mol (M_calc_: 8661.3725 g/mol).

## 4. Conclusions

Over the years the technique to synthesize peptides by SPPS has been optimized consistently. Today standard peptides can be obtained in good yields and purity. For drug development, peptides have to be synthesized under GMP conditions and to meet high quality criteria. Standard large peptides are difficult to produce in the desired quality. In light of these considerations the improvements obtained for the synthesis are remarkable.

Although the classical stepwise chain assembly of large peptides is complicated, we could show that the solid phase peptide synthesis described provides a suitable access to the 77-mer lipopeptide Myr-HBVpreS/2-78. The preliminary identification and assessment of difficult regions within the peptide chain by a predictive method helped to consider individual solutions. The incomplete coupling steps and resultant difficulties associated with the solid-phase synthesis of peptides containing difficult sequences were determined. Optimized chemical tactics reduced the occurrence of side products. An elevated reaction temperature could enhance the synthesis efficiency resulting in a product of higher yield and purity. Our data show that the chemical synthesis of the lipopeptide Myr-HBVpreS/2-78, the parent compound of novel peptides derived from the *N*-terminus of the large HBV envelope protein, is feasible by solid phase synthesis.
